# 患者报告结局在骨髓增生异常综合征中的应用

**DOI:** 10.3760/cma.j.issn.0253-2727.2022.08.019

**Published:** 2022-08

**Authors:** 琳琳 刘, 志坚 肖

**Affiliations:** 中国医学科学院、北京协和医学院血液病医院（中国医学科学院血液学研究所），实验血液学国家重点实验室，国家血液系统疾病临床医学研究中心，细胞生态海河实验室，天津 300020 State Key Laboratory of Experimental Hematology, National Clinical Research Center for Blood Diseases, Haihe Laboratory of Cell Ecosystem, Institute of Hematology & Blood Diseases Hospital, Chinese Academy of Medical Sciences & Peking Union Medical College, Tianjin 300020, China

骨髓增生异常综合征（MDS）常因外周血细胞减少相关的症状及体征而影响患者的健康相关的生活质量（HRQoL）[1]。较低危组MDS的主要治疗目标是改善外周血血细胞计数、提高生活质量，较高危组的主要治疗目标是延缓疾病进展和延长生存期[2]，因此，评估患者的HRQoL对于评估症状负担及疗效尤为重要。患者报告结局（PRO）作为评估患者生活质量的工具，是直接来自于患者对自身健康状况、脏器功能状态以及治疗感受的报告，不包括医务工作者、研究人员或其他任何人的解释或调整[3]，被认为是捕捉症状性不良事件的“金标准”[4]。现将PRO在MDS中的应用现况进行概述，以帮助我国医师提升对PRO在MDS诊治中应用的认识。

一、应用于MDS的PRO工具

（一）疾病特异性PRO工具

1. 针对癌症患者的PRO工具：欧洲癌症研究和治疗组织生活质量核心问卷（EORTC QLQ-C30）是使用最为广泛的评估癌症患者生活质量的工具之一，也是在MDS相关研究及临床试验中评估患者生活质量时应用最广泛的量表，其包含30个项目，得分为0到100分，得分越高表明生活质量越高[5]–[6]。该工具目前尚无MDS特异性疾病模块。近期，为了使EORTC QLQ-C30更适用于高危MDS、慢性粒单核细胞白血病和急性髓系白血病（AML）患者，Bell等[7]在EORTC QLQ-C30中增加了10个相关补充项目，提高了该量表在疲劳、呼吸急促和脏器功能方面的概念性覆盖。

癌症治疗功能评估-贫血（FACT-An）在有贫血症状的癌症患者中应用较多，其包含来自癌症治疗功能评估-常规（FACT-G）中的27个一般性项目、癌症治疗功能评估-疲劳（FACT-F）中的13个项目以及7个与贫血（非疲劳）相关的项目，分数越高表明生活质量越高[8]。已有研究结果证实了FACT-An在MDS人群中应用的可靠性和有效性[9]，Trudeau等[10]的研究也证明其在低危MDS群体中有很高的表面效度（Face validity）和内容效度（Content validity）。

血液恶性肿瘤患者报告结局（HM-PRO）是新近提出的一个针对血液恶性肿瘤患者的PRO评估工具，主要用于评估包括MDS等血液恶性肿瘤患者的HRQoL，其包含24个症状/体征相关问题的A部分及34个影响相关问题的B部分，并被验证具有很高的内部一致性及内容效度[11]。Goswami等[12]在905例患者中验证了其结构效度，且证实其与EORTC QLQ-C30和FACT-G都有较强的相关性。

2. 针对MDS的特异性PRO工具：骨髓增生异常综合征生活质量量表（QUALMS）是针对MDS的特异性量表，包含38个项目，分为躯体负担分量表（QUALMS-P）、有益发现分量表（QUALMS-BF）和情绪负担分量表（QUALMS-E），评分越高表明与MDS相关的生活质量越高[13]。该量表基于MDS患者和医护人员的访谈建立问卷。在前瞻性验证中，多中心的MDS队列完成了QUALMS、QLQ-C30及FACT-An量表，结果显示QUALMS量表内部一致性（α＝0.92）较高，重测信度较好（*r*＝0.81），且与QLQ-C30量表和FACT-An呈中等相关（|*r*|＝0.65～0.79，*P*<0.001），提示其较好的有效性和可靠性[14]。Trudeau等[10]亦在较低危MDS患者的横断面定性研究中证实该量表具有较强的表面效度和内容效度。

生活质量-E（QOL-E）是最早提出的MDS特异性PRO工具。量表包括一般健康状态、躯体、功能、社交、性、疲劳及MDS特异性问题等7个部分的29个项目，评分越高表明生活质量越高。其基于文献综述和对MDS患者及血液学家的访谈建立问卷。在试点和验证研究中，QOL-E的所有领域的内部一致性均显示良好（α>0.7），重测信度尚可（*r*＝0.65～0.80），主成分分析亦验证了该量表的结构效度，同时有效性验证显示QOL-E与FACT-G问卷同时评估有效性较好（*r*≥0.71）[15]。

（二）症状特异性PRO工具

疲劳作为QUALMS量表中最重要的一部分，是血液肿瘤的最常见症状[13]，因此评估疲劳程度的特异性PRO工具亦较为常用。最常用的疲劳相关量表为FACT-F。此外，20项多维度疲劳量表（MFI）、9项简要疲劳量表（BFI）也较为常用[5]。

焦虑、抑郁等心理症状同样在MDS患者中较为常见，针对心理功能评估，使用最多的量表是医院焦虑和抑郁量表（HADS），其次是情绪状态简表（POMS-SF）[5]。

（三）普适性PRO工具

普适性PRO工具即非特异针对某一症状或疾病的生活质量评估量表。常应用于MDS的普适性量表包括SF-36健康调查量表（SF-36）及其删减版本SF-12，除此之外，5项欧洲生活质量五维度量表（EQ-5D）和欧洲生活质量视觉模拟量表（EQ-VAS）亦有使用[16]。

二、临床实践中PRO工具的选用

由于PRO工具种类繁多，在临床应用以及设计临床研究时选择合适的工具至关重要且极具挑战。选用PRO工具时，除了将先前在相似患者分析中使用的问卷作为参考，还应重点考虑患者特点（如目标患者群体、所处疾病阶段、主要症状负担等）、问卷内容和问卷质量等问题[17]。成功的PRO问卷需要有明确的心理测量学研发过程和目标人群中的验证[18]，包括内部一致性、重测信度等信度分析以及内容效度、结构效度等效度分析，以及在患者病程中发生事件前后的反应性等验证试验。较好的信效度及反应性是选用该量表的前提，量表的语言版本、填写量表所需时间、量表研发及验证时的文化背景也需在应用前加以充分考虑。

目前用于MDS的PRO工具各自有其优点和潜在问题：症状特异性PRO工具可能更适用于评估患者某类特定症状负担，但其对总体生活质量的评估不够全面，也无法针对特定疾病针对性提取生活质量信息；EORTC QLQ-C30、FACT-An等癌症特异性工具及SF-36等普适性工具提出已久且得到了较充分的验证，现今在癌症领域应用非常广泛，但相对于MDS特异性工具，其描记MDS症状、涵盖临床结局方面不够精确；与此相对，MDS特异性工具能更精确地捕捉MDS患者的相关信息，但缺点在于还未在特定人群中得到足够的验证，充分验证后的MDS特异性PRO工具可能是低危MDS患者更好的选择[16]。

三、PRO在MDS中的具体应用

（一）PRO应用于疗效评估

提高生活质量是较低危组患者的治疗目标之一，因此评估治疗措施对生活质量的影响尤为重要。现今药物临床试验的主要结局聚焦于疾病稳定性、血液学反应、总体生存（OS）率及向AML转化的时间等，而PRO是从患者主观角度收集疾病状态以及治疗效果等信息的工具，将PRO纳入疗效评估有助于全面评估干预措施的效果和药物不良反应[19]。美国食品和药物管理局也认识到PRO的重要性，将减轻症状负担和改善HRQoL视为有效终点[20]，鼓励将PRO用于设计随机对照试验疗效的比较，并建议将其写入药物说明书以帮助医师能对治疗做出合理的决策[21]。因此，已有越来越多临床研究将PRO工具所评估的生活质量（QOL）作为次要研究终点。

1. 支持与促造血治疗：输血是MDS重要的支持治疗手段。Ryblom等[9]发现输血后7 d MDS患者的FACT-An总分中位数从50分增加到58分（*P*＝0.016），且发现FACT-An评分提高与HGB呈正相关（*r*＝0.66，*P*＝0.02）。然而，输血依赖可能会降低生活质量，Abel等[14]发现输血依赖与非输血依赖患者相比，QUALMS总体评分较低（62.4分对69.7分，*P*<0.01），提示输血依赖患者QOL更差。此外，Stanworth等[22]利用EORTC QLQ-C30和EQ-5D-5L评估采用严格输血阈值和自行决定输血阈值两种输血策略对生活质量的影响，结果显示后者取得生活质量提高的患者比例高于前者，自行决定组较限制组有更多患者表现出疲劳的改善（50％对30％），在随后分析中，自行决定组患者所有5项主要生活质量改善更为明显。

促红细胞生成素是治疗低危组MDS患者贫血的首选方法[23]。Fenaux等[24]报道EPO-α在低危组MDS患者中的有效性和安全性的随机、双盲、安慰剂对照临床试验，将QOL作为该研究的次要研究终点，利用FACT-An和EQ-5D-3L收集患者生活质量信息，结果显示24周时EPO-α组和安慰剂组获得治疗反应者之间EQ-5D指数得分有显著差异（*P*＝0.034），提示EPO-α可以提高MDS患者的生活质量。

血小板生成素受体激动剂艾曲波帕（Eltrombopag）可以改善血小板计数减少MDS患者的血小板计数和出血事件。一个单盲、随机、对照2期临床试验将血小板反应作为主要终点，将生活质量评分纳入次要终点，利用EORTC QLQ-C30和QOL-E评估患者生活质量。结果显示艾曲波帕组较安慰剂组有更高比例的患者出现血小板反应（*OR*＝27.1，95％*CI* 3.5～211.9，*P*＝0.0017）；在生活质量方面，患者QOL-E评分并未随时间出现显著差异，但QOL-E工具中社会、性、MDS特异性治疗结果指数、一般状态等项目分数随着血小板计数升高而有所提高[25]。

2. 免疫抑制剂与免疫调节剂治疗：Santini等[26]在一来那度胺治疗低危MDS患者的大系列、随机、安慰剂对照试验中用EORTC QLQ-C30量表评估来那度胺治疗对患者生活质量的改善，结果显示第24周时来那度胺治疗与减少疲劳（*P*＝0.046）和更好的情绪功能（*P*＝0.035）显著相关。调整基线后，来那度胺与更好的情绪功能相关（+0.8对−7.1，*P*＝0.047）。Revicki等[27]在来那度胺治疗中低危组MDS的随机、安慰剂对照3期临床试验中使用FACT-An量表评估来那度胺对患者生活质量的影响，结果显示自基线至治疗第12周，来那度胺5 mg/10 mg组与安慰剂组相比，FACT-An的总得分有所改善（+5.7/+5.7对−2.8，*P*值均<0.05），提示来那度胺治疗可有效改善患者HRQoL。

3. 去甲基化药物与化疗：去甲基化药物（HMA），特别是阿扎胞苷，是治疗高危MDS的一线药物。阿扎胞苷治疗MDS的3期临床试验使用EORTC QLQ-C30量表和心理健康量表（MHI）评估患者治疗过程中的生活质量变化，结果显示阿扎胞苷组患者在疲劳（EORTC，*P*<0.001）、呼吸困难（EORTC，*P*<0.0014）、身体功能（EORTC，*P*<0.0002）、积极情绪（MHI，*P*<0.0077）和心理困扰（MHI，*P*<0.015）方面较对照组评分显著提高[28]。Mittelman等[29]在阿扎胞苷联合来那度胺治疗高危组患者疗效的研究中，利用FACT-An量表评估该疗法对生活质量的影响，结果显示尽管只有部分患者的生活质量评分提高，但平均每例患者增加了5.1分，提示接受该治疗后患者生活质量略有改善。在CC-486（口服阿扎胞苷）的3期临床试验中，FACT-An被用于评估CC-486治疗对低危MDS患者生活质量的影响，结果显示治疗组与安慰剂对照组的FACT-An功能健康（FWB）评分皆较基线有所改善，但差异无统计学意义，而CC-486治疗组的试验结果指数（TOI）评分具有安慰剂组未出现的改善趋势[30]。

4. 异基因造血干细胞移植（allo-HSCT）：allo-HSCT是迄今唯一可望治愈MDS的方法，是治疗中、高危MDS患者有效方法[31]。Yu等[32]选用EORTC QLQ-C30问卷比较了低、中危MDS患者allo-HSCT与支持治疗/化疗对生活质量的影响，结果显示allo-HSCT组的患者生活质量较对照组有显著提高（53.8％对37.4％，*P*<0.05），提示中、低危MDS患者进行allo-HSCT对生活质量有益。Nakamura等[33]在较老年患者（50～75岁）中进行了减低强度预处理的allo-HSCT与去甲基化药物治疗/支持治疗的疗效比较，利用FACT-G、SF-36、EQ-5D等量表评估生活质量变化情况，结果显示移植组患者OS期和无白血病生存期显著高于非移植组，两组在生活质量评分都显著提高且有临床意义，但差异并无统计学意义。Patel等[34]在126例AML患者及84例MDS患者中比较白消安/环磷酰胺与白消安/氟达拉滨清髓预处理方案的效果，利用癌症治疗功能评估-骨髓移植量表（FACT-BMT）评估两组QOL并将其作为次要终点，结果显示两组生活质量评分组间差异无统计学意义。

（二）PRO用于患者预后评估

许多研究表明HRQoL与MDS患者OS率降低有关，因此应用PRO工具提取生活质量信息对于评估MDS患者预后有重要价值。Efficace等[35]利用EORTC QLQ-C30评估MDS患者疲劳程度，结果显示疲劳每增加10分与OS降低独立相关（*HR*＝1.11，95％*CI* 1.04～1.17，*P*＝0.0007）。Luskin等[36]的研究结果同样表明疲劳与OS降低独立相关（*HR*＝1.20，95％*CI* 1.01～1.41，*P*＝0.04），并提示严重的睡眠障碍对OS有更大的影响（*HR*＝2.95，95％*CI* 1.61～5.42, *P*＝0.002）。Troy等[37]发现心理痛苦水平升高与OS降低相关，该研究应用NCCN DT（Distress Thermometer）描记MDS患者心理痛苦水平，结果显示最大报告的DT≥4与较差的存活率有关（*P*＝0.01），在对诊断时的预后风险分层进行控制后，DT量表上每增加1分，死亡风险增加18％（*HR*＝1.18，95％*CI* 1.01～1.36，*P*＝0.03）。Buckstein等[38]则探究了包括脆弱性、共病、躯体功能等因素对MDS患者OS的影响，其利用洛克伍德CSHA 9点临床脆弱性量表（CFS）评估患者脆弱性程度，结果表明较严重的脆弱性是OS的独立相关因素（*HR*＝2.71，95％ *CI* 1.75～4.21，*P*<0.0001），躯体功能、疲劳、呼吸困难（QLQ-C30）以及总体健康状态（EQ-5D）也与OS显著相关。Starkman等[39]的研究同样发现脆弱性程度增加与OS率降低相关（*HR*＝2.76，95％*CI* 1.54～4.94，*P*＝0.0007）。

将生活质量信息与国际预后积分系统相结合可提高预后分层模型的预后能力。Luskin等[36]的研究结果表明增加疲劳信息使得仅包括IPSS-R、共病指数和性别的标准生存模型的区分度稍有提高（标准模型、标准模型+疲劳严重度、标准模型+睡眠障碍的bootstrap校正后C-index分别为0.701、0.702、0.704）。Efficace等[40]提出了一个新的预后积分系统FA-IPSS（h），把EORTC QLQ-C30疲劳量表45分作为分界值，将IPSS中危-2组和高危组患者重新分为了3组：Risk-1为IPSS中危-2伴低度疲劳（<45分），Risk-2为IPSS中危-2伴高度疲劳（≥45分）或IPSS高危伴低度疲劳，Risk-3为IPSS高危伴高度疲劳，其中位OS期分别为23、16和10个月，并发现Risk-2（*HR*＝1.694，95％*CI* 1.243～2.307，*P*<0.001）和Risk-3（*HR*＝3.123，95％*CI* 1.787～5.456，*P*<0.001）与Risk-1相比OS预后更差。该积分系统较IPSS有更好的拟合优度（AIC分别为1118.0、1122.6）和更高的准确率（C-index分别为0.581、0.535）。Buckstein等[38]发现，将CFS评估的脆弱性信息结合IPSS-R的预后模型相较于IPSS-R，其区分度提高了30％（综合判别改善指数分别为20％、26％），并将原有的分组重新划分为3个风险组：0～1（中位生存期未达到）、2～3（中位生存期32个月）和4（中位生存期4个月）。Starkman等[39]提出了一个MDS特异性脆弱性指数（MDS-FI），其将年龄、FI与IPSS、输血依赖情况四项内容结合起来构建了MDS-FI，结果显示该新积分系统区分度较IPSS和IPSS-R高（C-index分别为74％、67％、67％），FI、年龄和输血依赖状况对IPSS的综合判别改善增量分别为37％、11％和5％，利用IPSS-R、FI以及年龄构建的另一系统的综合判别改善增量为18％，提示将脆弱性指数纳入预后分层系统相较传统预后系统提高了预测精度，对MDS患者生存期的预测及治疗方案的选择具有一定的参考价值。

四、小结与展望

患者报告结局注重患者主观感受，帮助我们更全面地评估患者症状负担、治疗效果以及预后。PRO的重要性和有效性也因此日益得到认识，越来越多研究应用PRO工具评估患者生活质量[41]。但在目前MDS相关研究及临床试验中，应用了PRO工具的仍是少数，针对MDS的特异性工具则应用得更少[5]。这表明PRO工具尚未得到充分利用，尽管有多种用于评估HRQoL的有效工具，但针对MDS患者的特异性工具有限，且目前提出的特异性工具尚需多中心大系列研究来加以验证。此外，PRO是否可以作为预后工具这一问题亦需在更一致的患者群体中进一步进行确证研究[41]。在日常临床实践中，填写问卷对患者造成的负担也需要研究者及临床医师多加考虑，选用最适合目标人群的PRO工具也仍是一项挑战。当特异性工具的可靠性、有效性和反应性在目标人群中得到证明，计算机自适应系统等新技术被应用于量表的填写，PRO工具在未来的MDS研究中定将得到更广泛的应用。
